# Treatments for reversing warfarin anticoagulation in patients with acute intracranial hemorrhage: a structured literature review

**DOI:** 10.1186/1865-1380-4-40

**Published:** 2011-07-08

**Authors:** Brett F Bechtel, Timothy C Nunez, Jennifer A Lyon, Bryan A Cotton, Tyler W Barrett

**Affiliations:** 1 Department of Emergency Medicine, Vanderbilt University Medical Center, Nashville, TN, USA; 2 Eskind Biomedical Library, Vanderbilt University School of Medicine, Nashville, TN, USA; 3 Department of Surgery, Brooke Army Medical Center, Fort Sam Houston, TX, USA; 4 Department of Surgery, Center for Translational Injury Research/The University of Texas Medical School, Houston, TX, USA

## Abstract

**Study objective:**

The acute management of patients on warfarin with spontaneous or traumatic intracranial hemorrhage continues to be debated in the medical literature. The objective of this paper was to conduct a structured review of the medical literature and summarize the advantages and risks of the available treatment options for reversing warfarin anticoagulation in patients who present to the emergency department with acute intracranial hemorrhage.

**Methods:**

A structured literature search and review of articles relevant to intracranial hemorrhage and warfarin and treatment in the emergency department was performed. Databases for PubMed, CINAHL, and Cochrane EBM Reviews were electronically searched using keywords covering the concepts of anticoagulation drugs, intracranial hemorrhage (ICH), and treatment. The results generated by the search were limited to English- language articles and reviewed for relevance to our topic. The multiple database searches revealed 586 papers for review for possible inclusion. The final consensus of our comprehensive search strategy was a total of 23 original studies for inclusion in our review.

**Results:**

Warfarin not only increases the risk of but also the severity of ICH by causing hematoma expansion. Prothrombin complex concentrate is statistically significantly faster at correcting the INR compared to fresh frozen plasma transfusions. Recombinant factor VIIa appears to rapidly reverse warfarin's effect on INR; however, this treatment is not FDA-approved and is associated with a 5% thromboembolic event rate. Slow intravenous dosing of vitamin K is recommended in patients with ICH. The 30-day risk for ischemic stroke after discontinuation of warfarin therapy was 3-5%. The risks of not reversing the anticoagulation in ICH generally outweigh the risk of thrombosis in the acute setting.

**Conclusions:**

Increasing numbers of patients are on anticoagulation including warfarin. There is no uniform standard for reversing warfarin in intracranial hemorrhage. Intravenous vitamin K in addition to fresh frozen plasma or prothrombin complex concentrate is recommended be used to reverse warfarin-associated intracranial hemorrhage. No mortality benefit for one treatment regimen over another has been shown. Emergency physicians should know their hospital's available warfarin reversal options and be comfortable administering these treatments to critically ill patients.

## Introduction

Outpatient prescriptions for warfarin increased 45% to 31 million in the United States during the period of 1998-2004 [1]. Warfarin usage will increase with the rising prevalence of diseases such as atrial fibrillation and the aging of the population [2]. There are more than 1 million emergency department (ED) visits annually for traumatic brain injury (TBI) in the US [3]. The use of warfarin increases a patient's risk for spontaneous intracranial hemorrhage and overall mortality. The incidence of spontaneous intracranial hemorrhage (ICH) is 7-10 times higher among patients taking warfarin compared to those not on anticoagulation [4]. Patients taking warfarin therapy account for 8-14% of all ICH [4,5], and ICH occurs 8-10 times more frequently in individuals on warfarin [4,6]. The annual risk of ICH in patients treated with warfarin has been estimated to be between 0.3-2.0% [7-10]. ICH is the most lethal form of CVA with 30-day mortality rates estimated at 30-55% [8,9,11], while those on warfarin have a higher risk of death at 30 days of 43-60% [9,11,12]. Alarmingly, between 50-90% of all ICH occurs while the INR is within the target range [7].

Different therapies such as fresh frozen plasma (FFP), vitamin K, prothrombin complex concentrates (PCC), which contain factors II, VII, IX, and X, and recombinant activated factor VII (rfVIIa) have all been used as ways to treat ICH in this high-risk population, either alone or in combination. Acute interventions that minimize or arrest ongoing bleeding and hematoma size are likely to be critical for improving outcomes [4,13]. This makes the role of the ED physician paramount in diagnosis and management of warfarin-associated ICH in a timely manner.

The objective of this systematic review is to summarize the medical literature regarding the benefits and risks of the available treatment options to reverse warfarin anticoagulation in patients with acute ICH.

## Materials and methods

### Study design and data sources

We conducted a comprehensive literature search of the databases MEDLINE^® ^(via PubMed^®^; 1950-) through 2009 using a combination of Medical Subject Terms (MESH^®^) and keywords covering the concepts of anticoagulation drugs, intracranial hemorrhage (ICH), and treatment. A sample search strategy was "(Anticoagulants[mh] OR Anticoagulants [Pharmacological Action]) AND Intracranial Hemorrhages[mh] AND therapeutics[mh] AND English[la]," which yielded 1,082 citations as of 29 December 2009. We limited the search to study designs that included the following: clinical trial, meta-analysis, and practice guideline, randomized controlled trial. This search resulted in a total of 382 (/29 December 2009) abstracts.

Two independent reviewers (BFB, TCN) examined the abstracts and made separate recommendations for inclusion. A third reviewer (TWB) adjudicated any disagreements between the two primary reviewers. This review yielded 37 articles that were selected for full-text evaluation based on the following inclusion/exclusion criteria:

1. Patient taking an anticoagulant drug, with preference for warfarin

2. Patient diagnosed with intracranial hemorrhage

3. Article contains information on treatment/management of ICH

4. Article contains raw data and was original research study

From these, 14 additional articles were hand selected from the papers' references for inclusion. This yielded a total of 51 studies, of which 30 were reviews. The reviews were excluded, resulting in 21 original studies for evaluation.

We additionally searched the Cochrane Database of Systematic Reviews (via OVID^®^) and the Cumulative Index of Nursing and Allied Health Literature (CINAHL^® ^Via EBSCOhost^®^) for relevant reviews and articles as of December 2009. We identified no relevant reviews in the Cochrane Database. A search of CINAHL--using ((MH "Intracranial Hemorrhage+") or (MH "Cerebral Hemorrhage+")) AND ((MH "Anticoagulants+") or (MH "Heparin+") or (MH "Warfarin"))--limited to English language, Clinical Trials, Journal Articles, Practice Guidelines, Research, and Systematic Reviews, retrieved 204 abstracts of which 31 were selected. Three of the 31 were eliminated as duplicates to the PubMed search, yielding 28 that were added to the PubMed results for further examination. Two independent reviewers (BFB, TWB) examined the 28 abstracts and chose two for ultimate inclusion in the paper based on the above inclusion/exclusion criteria. The final consensus of our comprehensive search strategy was a total of 23 original studies for inclusion in our review. Two reviewers assessed the quality of evidence for each of these 23 manuscripts using the GRADE system that classifies studies from high to very low based on study design and internal validity measures [14].

## Results

The available literature consists primarily of small case series and retrospective cohort studies with the majority classified as low, thus limiting the strength of findings [14]. Variation in federal regulatory drug agency approvals also impacts reporting as PCC is approved and considered a standard reversal treatment in most nations [6]. The US Federal Drug Agency, however, has yet to approve a PCC formulation that is sufficient for warfarin reversal, thus leaving thawed plasma as the only available clotting factor option for rapid reversal.

This review focuses on warfarin-associated intracranial hemorrhage (WAICH) from spontaneous causes; however, the acute management of WAICH appears to include the same treatments regardless of whether the ICH was spontaneous or traumatic. However, the literature on traumatic WAICH is limited because of small sample sizes (Table 1) [15-18].

**Table 1:** Studies investigating reversal of warfarin anticoagulation in traumatic intracranial hemorrhage

Author	Study type	Patient population	Intervention	Result	**Grade **[14]
Bartal et al. 2007	Prospective	7 patients on warfarin with traumatic ICH	All received vitamin K and 6-12 units of FFP. INR was still > 1.3 in all, so 40-90 μg/kg rfVIIa was given. INR dropped below 1.3, and all underwent neurosurgery	rfVIIa lowered the INR into operable range in all patients.	Low
Baldi et al. 2006	Prospective	232 on warfarin or acenocumarol or with INR equal or > 2 with spontaneous or traumatic ICH	FFP used in 22% of patients, vitamin K in 41%, PCC in 6% and factor VII concentrate in 3%. Many did not receive any reversal treatment	No statistical differences were found in the outcomes of patients with or without medical therapy	Low
Kalina et al. 2008	Prospective	46 trauma patients on warfarin with ICH with INR > 1.5	Institution developed protocol for trauma patients with ICH taking warfarin with INR > 1.5. Patients given weight-based dose of PCC (concentrated II, VII, IX, X). All given 5 mg vitamin K as well. Patients could receive FFP as well in protocol group	Protocol resulted in increased number of patients receiving PCC. Protocol patients had improved times to INR normalization, patients having reversal of coagulopathy, and shorter times to surgery. No difference in ICU days, hospital days, or mortality. 2/48 that got PCC had DVT	Low
Ivascu et al. 2006	Retrospective	57 patients with traumatic WAICH from fall or MVC	Established and implemented protocol to treat traumatic WAICH. All 35 protocol patients received FFP. Only 14/22 patients in the pre-protocol group received FFP	Mortality and reversal times of INR were similar between the protocol instituted group and the pre-protocol group	Low

### Rationale for rapid correction of anticoagulation in the emergency department

Warfarin not only increases the risk of but also the severity of ICH by causing hematoma expansion [10]. Hematoma progression was found to occur in nearly 40% of ICH patients with ICH in the first few hours following symptom onset [4]. Hematoma enlargement within 6-12 h is commonly seen in patients on warfarin with ICH [19]. As volume and ventricular extension increase, earlier patient deterioration is observed [20]. Goldstein et al. found that for every 30-min delay in FFP administration, the probability of successful INR reversal within 24 h decreases by 20% [21]. Interventions aimed at preventing this growth are paramount as larger hematomas are associated with poorer functional outcomes [13,20].

### Administration of prothrombin complex concentrates versus fresh frozen plasma

Warfarin inhibits the production of vitamin K-dependent clotting factors; therefore, patients with WAICH should have their clotting factors repleted. Treatment options for the repletion of clotting factors include FFP and PCC.

FFP contains all coagulation factors in non-concentrated form. FFP is more universally available at hospitals, especially in the US, compared to PCC. Administration of FFP, a blood product, requires compatibility testing and carries the risk of blood borne infection transmission and transfusion-related acute lung injury (TRALI). FFP is stored frozen, thus requiring at least 15-20 min to thaw [6]. Large FFP volumes (800 to 3,500 mL) are often needed to treat serious hemorrhages [22]. This large volume may result in acute decompensated heart failure in patients with atrial fibrillation or cardiac valve disease and ventricular dysfunction. Less common adverse events including allergic reactions are also possible with FFP [6].

PCC contains coagulation factors II, VII, IX, and X, and proteins C, S, and Z in concentrated form [6]. European and Australian practice protocols recommend the use of PCC in bleeding emergencies [6]. PCC is not widely available in US hospitals, and, while cheaper than rfVIIa, it is more expensive than FFP. PCC risks include potential thrombotic complications and disseminated intravascular coagulation (DIC) [6]. The optimal PCC dosing is calculated according to patient age, body weight, severity of INR prolongation, and desired level of INR correction with typical dosages of 25 to 50 IU/kg [19].

Studies have compared the use of PCC and FFP in patients with WAICH, measuring which treatment corrected the INR faster (Table 2). Makris et al. found that PCC outperformed FFP at repleting factors II, VII, IX, and X [23]. PCC administration normalized the INR, whereas the INR remained elevated in patients given FFP [23]. Fredriksson et al. retrospectively found that PCC worked significantly faster than FFP. The mean INR decreased from 2.83 to 1.22 within 4.8 h in the ten patients treated with PCC compared with an INR decrease from 2.97 to 1.74 within 7.3 h in those seven patients receiving FFP [24]. These findings, however, are not surprising as FFP lacks concentrated factors and has an INR of 1.3-1.4.

**Table 2:** Studies investigating PCC versus FFP for anticoagulation reversal in warfarin-associated intracranial hemorrhage

Author	Study type	Patient population	Intervention	Result	**Grade **[14]
Makris et al. 1997	Prospective	16 patients with WAICH, along with 12 "similar subjects"	Vitamin K 1-5 mg IV given to all patients. 16 patients got PCC and 12 FFP	PCC repleted factors II, VII, IX, and X better than FFP. In patients given FFP, INR remained elevated. 28/29 patients given PCC had INR correction	Moderate
Fredriksson et al. 1992	Retrospective	17 patients with WAICH	All patients received vitamin K 10-20 mg IV. Of the 17 total patients, 10 received PCC and 7 received FFP	PCC significantly decreased the INR from 2.83 to 1.22 within 4.8 h, compared with a decrease in INR from 2.97 to 1.74 within 7.3 h in the FFP group. Signs and symptoms of ICH progressed more in those treated with FFP than with PCC	Low
Boulis et al. 1999	Prospective, randomized controlled trial	13 patients with WAICH	All patients received vitamin K 10 mg subcutaneously. 8 patients received FFP. 5 patients received weight-based dosing of factor IX complex concentrate (FIXCC) in addition to FFP	Significant differences were found in time to correction, rate of correction, and volume of FFP required for correction between the FFP group (8.9, 2,700 mL) and the FIXCC + FFP group (2.95, 399 mL)	Moderate
Cartmill et al. 2000	Prospective	12 patients with spontaneous WAICH	6 patients treated with 50 μg/kg IV PCC along with vitamin K 10 mg IV. 6 matched patients treated with 4 units of FFP and vitamin K 10 mg IV. INR re-checked 15 min after treatment	PCC group had significantly faster and complete reversal compared to the FFP group. Mean post-treatment INRs were 1.32 in PCC group and 2.3 in FFP group	Low
Siddiq et al. 2008	Retrospective	19 patients with diagnosis of WAICH	10 patients treated with PCC, vitamin K, and FFP, and 9 patients treated with FFP and vitamin K	PCC along with FFP and vitamin K trends toward faster normalization of INR than with FFP and vitamin K alone	Low

Cartmill et al. prospectively studied 12 patients with WAICH. They treated six patients with 50 μg/kg intravenous (IV) PCC and 10 mg IV vitamin K. Six matched patients were treated with 4 units of FFP and 10 mg IV vitamin K. The investigators measured the INR at baseline and 15 min after treatment. The PCC group had significantly faster and complete reversal compared to the FFP group. The mean post-treatment INRs in the PCC and FFP groups were 1.32 and 2.3, respectively [25].

Boulis et al. performed a randomized control trial comparing treatment with FFP and FFP + factor IX complex concentrate (FIXCC) in patients with acute ICH. FIXCC, similar to other PCC formulations, contains high concentrations of activated vitamin K-dependent factors (factors II, VII, IX, and X). The study included 13 patients, 8 receiving FFP alone and 5 receiving FFP and FIXCC. All patients received vitamin K 10 mg subcutaneously (SQ). They reported significant differences in time to correction, rate of correction, and volume of FFP required for correction between the FFP group (mean 8.9 h, 2,700 cc) and the FIXCC + FFP group (mean 2.95 h, 399 cc) [22]. Another study retrospectively evaluated ten patients treated with FIXCC + FFP + vitamin K and nine patients treated with FFP + vitamin K alone [26]. FIXCC used along with FFP and vitamin K trends toward faster normalization of INR than with FFP and vitamin K alone.

In conclusion, PCC is statistically significantly faster than FFP at correcting the INR in patients taking warfarin. None of these studies, however, demonstrated a statistically significant clinical outcome difference between those treated with FFP or PCC.

### Use of recombinant factor VIIa for warfarin reversal in ICH (Table 3)

**Table 3:** Studies investigating recombinant factor VIIa for anticoagulation reversal in intracranial hemorrhage

Author	Study type	Patient population	Intervention	Result	**Grade **[14]
Bartal et al. 2007	Prospective	7 patients on warfarin with traumatic ICH	All received vitamin K and between 6- 12 units of FFP. INR was still > 1.3 in all and so 40-90 μg/kg rfVIIa was given. INR went below 1.3, and all underwent neurosurgery	The use of rfVIIa lowered the INR into operable range in all patients	Low
Sorensen et al. 2003	Retrospective	6 patients with WAICH	All received vitamin K, three received FFP. INR still > 1.5 so 10-40 μg/kg rfVIIa given to each patient. All underwent NSGY	All INRs were equal to or < 1.5 within 10 min of rfVIIa being given and allowed for safe neurosurgical procedure	Very low
Freeman et al. 2004	Retrospective	7 patients with symptomatic non-traumatic WAICH	Treated with 15-90 μg/kg of rfVIIa. Vitamin K given to all patients as well except for one who died prior. All patients but one also received FFP. Two underwent neurosurgical procedures	IV bolus rfVIIa rapidly lowered the INR to normalized levels	Very low
Brody et al. 2005	Retrospective	28 patients with WAICH with INR > 1.3	15 patients received 10 mg IV or subcutaneously vitamin K and FFP. 12 patients received vitamin K, FFP, and rfVIIa as well	Median time from presentation to INR < 1.3 was 8.8 h in the rfVIIa group and 32 h in the FFP group. Significantly lower. One patient with ESRD developed DIC after three doses of rfVIIa. One patient in the FFP group developed pulmonary edema	Low
Nishijima et al. 2010	Retrospective	40 patients with traumatic WAICH and INR 1.3 or greater	20 patients received rfVIIa and 20 did not. In both groups some patients received pRBCs, FFP, and vitamin K. Patient characteristics were similar in both groups	No difference in mortality. 7 patients died in each group. There were 4/20 thrombotic complications in the rfVIIa group and 1/20 in the control. This was not statistically significant. Time to INR normalization was faster in the rfVIIa cohort mean = 4.8 h than in the standard cohort mean = 17.5 h	Low

Recombinant factor VIIa (rfVIIa) is the cloned activated form of endogenous human hemostatic factor VII. Its original use was for hemophiliac patients. rfVIIa is given as an IV bolus over 2-5 min, with its onset of action being almost immediate and clinically apparent hemostasis observed within 10 min [27]. However, it is expensive to use, with a 1.2-mg vial costing approximately USD$1,369 [27]. There is no risk of blood-borne pathogen transmission, but there is a clinically important risk of thrombotic complications. A systematic review of 35 randomized clinical trials reported arterial and venous thromboembolic rates of 5.5% and 5.3%, respectively [28]. The half-life of rfVIIa is short unless the dose is increased to 120 μg/kg. At this dose, rfVIIa was able to correct INR for periods of 24 h without signs of systemic coagulation in one study [27].

Bartal et al. in 2007 did a prospective study on seven patients with WAICH. They all received vitamin K and between 6- 12 units of FFP. The INR was still greater than 1.3 in all, and so 40-90 μg/kg IV rfVIIa was given. This rapidly corrected the INR to below 1.3 [15]. Sorensen et al. evaluated six patients who all received vitamin K, and three were transfused FFP. The patients' INRs remained elevated (> 1.5), so each patient was given 10-40 μg/kg rfVIIa in preparation for emergent neurosurgery. All six patients' INRs were ≤ 1.5 within 10 min of rfVIIa administration and underwent neurosurgery [29]. Freeman et al. treated seven patients with 15-90 μg/kg of rfVIIa. All patients but one also received vitamin K and FFP. They found that an rfVIIa IV bolus rapidly lowered the INR to normalized levels [30]. Brody treated 15 patients with vitamin K 10 mg IV or SQ and FFP. Twelve patients received vitamin K, FFP, and rfVIIa as well. Median time from presentation to INR < 1.3 was significantly lower (*p *= 0.016) in the rfVIIa group (8.8 h) compared with the FFP group (32 h). One patient with ESRD developed DIC after three doses of rfVIIa. One patient in the FFP group developed pulmonary edema [31].

Nishijima et al. retrospectively analyzed 40 patients with WAICH including 20 who received rfVIIa. They found no difference in mortality. Seven patients died in each group. Four of the 20 thrombotic complications occurred in the rfVIIa group and in the control group (*p *> 0.05). Time to normalization of INR was significantly faster (*p *< 0.001) in the rfVIIa cohort (mean = 4.8 h) versus the standard cohort (mean = 17.5 h) [32].

In conclusion, recombinant factor VIIa appears to rapidly reverse warfarin's effects on INR. This is not an FDA-approved use. This potential rapid reversal benefit must be weighed against the reported 5% risk of a thromboembolic event.

### Is vitamin K safe and effective and how should it be given?

Vitamin K is a necessary component within the liver in order to help carboxylate factors II, VII, IX, and X to their active forms. When warfarin is given, it blocks the reductase that converts oxidized vitamin K back to vitamin K for reuse [4]. A vitamin K shortage within the liver creates a coagulation cascade deficit. Vitamin K replacement in patients on warfarin can be administered by oral, SQ, or IV route. Oral and SQ routes have variable times to onset and slower absorption rates. Intravenous dosing is recommended in those patients with ICH. Dosages of 10 mg given by slow IV infusion over 30 min has been recommended [11]. The time to onset is at least 2-6 h and often more than 24 h are needed to achieve effective response [4]. Other factor replacement must be given in the interim as well. The incidence of anaphylactic reaction to IV vitamin K is exceedingly rare with a reported rate of 3 out of 10,000 doses [4]. Many of the reported anaphylaxis episodes occurred with older vitamin K formulations containing polyethoxylated castor oil, while the modern micelle formulation is thought to have a lower risk of anaphylactoid reactivity [33]. A 2001 review of anaphylactoid reactions associated with vitamin K reported 23 (3 fatal) case reports and 132 FDA-reported adverse drug events from IV vitamin K, 32 individuals following intramuscular administration, 13 patients following SQ vitamin K, and 7 individuals following oral vitamin K [34]. In conclusion, 5-10 mg of vitamin K should be given slowly by IV when a patient presents with WAICH.

### What is the risk of thrombosis after warfarin reversal?

Patients who take warfarin for atrial fibrillation, pulmonary embolus, deep vein thrombosis, or mechanical heart valves are at risk for thrombotic or embolic risk when their anticoagulation is reversed. Physicians need to consider this risk when deciding to reverse the anticoagulation. Most reviews and studies view it as an acceptable risk for reversal in the setting of warfarin ICH [7]. Phan et al. showed that discontinuation of warfarin therapy for 1 to 2 weeks in patients with a high embolic risk is relatively safe [35]. The risk for ischemic stroke after discontinuation of warfarin therapy within 30 days was 3% for metallic valves, 3% with atrial fibrillation, and 5% in those with recurrent TIA or minor stroke [35]. Once warfarin is restarted, early recurrence of ICH is exceedingly uncommon [35]. In conclusion, treating physicians should correct the INR no matter the reason the patient is on anticoagulation. The risk of not treating the ICH and lowering the INR generally outweighs the risk of thrombosis in the acute setting.

### Does rapid correction of anticoagulation improve patient mortality?

Numerous small studies (Table 4) have reported that the warfarin reversal agents result in more rapid INR normalization and decreased intracranial hematoma expansion [36-40]. The medical literature's reporting of these reversal agents' impact on patient survival is limited to relatively small, primarily retrospective, studies that have investigated whether treatment with a single agent, combination therapy of FFP, vitamin K, PCC, or no reversal therapy affects patient mortality (Table 5) [41-43]. Berwaerts et al. studied 68 patients with WAICH and 126 not on warfarin with ICH between 1993-1999 [41]. The treatment of the 68 patients on warfarin consisted of the following: 19 received only vitamin K, 11 vitamin K and FFP, 5 FFP alone, 3 vitamin K and FFP and factor IX, 2 vitamin K and factor IX, 2 factor IX alone, and 26 received no therapy and were treated with an "expectant attitude." Overall the patients in the study who were not on warfarin had an inpatient mortality of 18% versus 38% of those on warfarin [41]. The authors reported no difference in mortality among patients with WAICH who had been reversed with any combination or had not been reversed at all. This method of retrospectively assessing treatment effect is confounded by a great number of treatment arms and small number of patients.

**Table 4:** Studies investigating multiple treatment options for anticoagulation reversal in warfarin-associated intracranial hemorrhage

Author	Study type	Patient population	Intervention	Result	**Grade **[14]
Rabinstein and Wijdicks 2007	Retrospective	13 patients with spontaneous WAICH	Vitamin K and FFP in "doses deemed appropriate for each case." Neurosurgical intervention once INR < 1.4	Median time to reversal 6.5 h (INR < 1.4). Recovery in 65% of those patients who fully awoke within 36 h after evacuation	Low
Yasaka et al. 2005	Prospective	35 patients with WAICH	Varying doses of PCC (200- 1,500 IU) were given to see what the optimal dose was for INR correction	200 IU did not decrease 50% of the patients below 2.0 INR. 500 IU decreased the INR to < 1.5 in 96% of patients with initial INR < 5.0. All patients treated with 1,000 IU-1,500 IU had INR decrease to < 1.3	Low
Preston 2002	Prospective	10 patients with WAICH	PCC dose range 25-50 μg/kg was used in each patient as reversal as well as vitamin K 2-5 mg IV	Median INR was 3.98 prior to treatment and 20 min after treatment < 1.9 with almost all < 1.3	Low
Nitu et al. 1998	Retrospective	1 patient with WAICH; 17 patients on warfarin with other bleeding	Factor IX and factor VII concentrate given to patients	INR in the patient with ICH went from 5.9 to post-treatment 1.8 within 15 min	Low
Lee et al. 2006	Retrospective	45 patients with WAICH	Varying doses of FFP and vitamin K were given for reversal	The median time for door to INR normalization was 30 h (14 to 49.5), with 4 patients' hematomas enlarging after INR normalization	Low

**Table 5:** Studies investigating the available anticoagulation reversal agents' impact on patient survival

Author	Study type	Patient population	Intervention	Result	**Grade **[14]
Berwaerts et al. 2000	Retrospective	68 patients with WAICH	19 patients received vitamin K only, 11 vitamin K + FFP, 5 FFP, 3 vitamin K + FFP + factor IX, 2 vitamin K + factor IX, 2 factor IX, and 26 were treated with an "expectant attitude"	No significant difference in mortality rate among patients who had been reversed with any combination of reversal agents or had not been reversed	Low
Sjoblom et al. 2001	Retrospective	136 patients with WAICH who received some form of reversal	Either single therapy or combinations of vitamin K, factor IX, FFP, or no therapy was administered	No evidence that any treatment strategy was superior to the others	Moderate
Huttner et al. 2006	Retrospective	55 patients with WAICH	Compared vitamin K, PCC, and FFP alone or in some combination	Incidence and extent of hematoma growth were significantly lower in the PCC-treated group. If the INR was normalized within 2 h then FFP and PCC influence on hematoma growth were similar	Low
Goldstein et al. 2006	Retrospective	69 patients with non traumatic WAICH with INR > 1.4	Patients received no therapy, FFP, vitamin K, or combination	Patients whose INR was successfully reversed within 24 h had a shorter time from diagnosis to first dose of FFP (90 vs. 210 min). Shorter time to vitamin K as well predicted INR correction. Every 30 min of delay in the first dose of FFP was associated with 20% decreased odds of INR reversal within 24 h. No ED intervention was associated with improved clinical outcome	Moderate
Yasaka et al. 2003	Prospective	15 patients with WAICH	9 PCCs with vitamin K 10-20 mg IV, 2 PCCs alone or 4 with vitamin K 10-20 mg IV alone were administered based on decision of treating MD	Vitamin K lowered the INR after 12-24 h to normalized range. PCC with or without vitamin K was more effective at rapidly correcting the increased INR. PCC without vitamin K administration led to a recurrent increase in INR after 12-24 h	Low

Sjoblom et al. performed a similar study in 2001, retrospectively reviewing charts of 136 patients with WAICH between 1993-1996, who all received some form of reversal [20]. Either single therapy or combinations of vitamin K, factor IX, FFP, or no therapy was administered. There was no evidence that any treatment strategy was superior to the other [20]. Huttner et al. looked at 55 patients with WAICH and compared vitamin K, PCC, and FFP alone or in some combination. They found the incidence and extent of hematoma growth were significantly lower in the PCC-treated group [42]. If the INR was normalized within 2 h, then the influences of FFP and PCC on hematoma growth were similar [42].

In conclusion, multiple small retrospective studies with many treatment arms did not find any difference in one treatment over another or no treatment at all in correcting the INR of those patients with WAICH [20,21,41,42]. However, these may very well reflect a type II error given their underpowered sample sizes. A large well-designed prospective study is still needed to determine whether rapid correction of anticoagulation is effective at improving patient-oriented outcomes (i.e., functional neurologic recovery) and an appropriate utilization of healthcare resources.

## Conclusions

Emergency medicine physicians will see increasing numbers of patients on warfarin as the population ages. Physicians must know how to treat patients who present with warfarin-associated intracranial hemorrhage. PCC appears to normalize the INR faster than FFP. Vitamin K is generally safe and should be administered by slow IV infusion in all patients who present with WAICH. rfVIIa has not yet been approved for use in WAICH, but has shown promising results in fast normalization of INR in a small subset of patients, but at the cost of a 5% thromboembolic event rate. Despite the multiple treatment options to correct anticoagulation, studies have yet to demonstrate improved patient survival with any particular treatment strategy.

## Funding sources

No industry financial support or compensation was received for conducting this study. The study was entirely funded by the Vanderbilt University Medical Center Department of Emergency Medicine Research Division. Dr. Barrett is supported in part by NIH grant K23 HL102069 from the National Heart, Lung, and Blood Institute.

## Disclosure

Dr. Cotton currently serves as an adjudicator for ongoing research sponsored by CSL Behring (makers of prothrombin complex concentrate).

## Competing interests

The other authors declare that they have no competing interests.

## Authors' contributions

TB and BC conceived the study idea. TB, BB, BC, TN, and JL developed the study design and objectives. JL queried numerous medical literature databases on multiple occasions to retrieve the most current literature on the study topic. BB, TN and JL performed the initial screening of potentially eligible abstracts. TB adjudicated disagreements in potential article eligibility. BB wrote the initial draft of the paper. BB, TN, JL, TN, BC, and TB directly participated in the multiple revisions of the intellectual content of the manuscript. All authors read and approved the final manuscript
